# A Rare Case of Traumatic Pericardio-Diaphragmatic Injury Following a Road Traffic Accident

**DOI:** 10.7759/cureus.39125

**Published:** 2023-05-17

**Authors:** Rajeev Thilak Chellasamy, Nembian Rajarajan, Noel Marie Pio Samy, Hemachandren Munusamy

**Affiliations:** 1 Cardiac/Thoracic/Vascular Surgery, Jawaharlal Institute of Postgraduate Medical Education & Research, Puducherry, IND; 2 Anaesthesiology, Jawaharlal Institute of Postgraduate Medical Education & Research, Puducherry, IND; 3 Cardiac/Thoracic/Vascular Surgery, Jawaharlal Institute of Postgraduate Medical Education & Research, Pondicherry, IND

**Keywords:** traumatic diaphragmatic hernia, blunt abdominal injury, pericardial injury, diaphragmatic rupture, major trauma

## Abstract

Traumatic pericardi0-diaphragmatic rupture is very rare. It occurs due to high-velocity blunt trauma or penetrating injury to the abdomen or chest, which requires emergency intervention. The severity of injury varies, and it is very difficult to diagnose. Diaphragmatic ruptures are more common on the left side. Pericardial tears and diaphragmatic rupture are rare and often unrecognized in the acute phase. Computed Tomography is essential to diagnose and requires emergency surgery to avoid dreaded complications. A twenty-eight-year-old female reported to the emergency department with blunt injury to the abdomen following a road traffic accident. She was found to have diaphragmatic and a pericardial rupture with herniation of the bowel into the thoracic cavity. Emergency surgical repair was done. We report this case, as it is very rare to have a pericardial involvement along with diaphragmatic rupture, and to elaborate on the technical aspect of the surgical repair.

## Introduction

Traumatic diaphragmatic rupture usually results from high-velocity blunt trauma to the abdomen. The incidence of pericardial rupture and diaphragmatic injury is 0.2-3.3% [1]. Pericardial rupture creates communication between the peritoneum and the pericardial cavity. They are most often associated with other abdominal visceral injuries [2]. It is a marker of severe traumatic injury [3]. These type of injuries are life-threatening and requires immediate surgical repair, and it is difficult to diagnose pre-operatively. We report a twenty-eight-year-old woman who sustained pericardio-diaphragmatic rupture following blunt abdominal trauma. She was taken up for emergency surgery and was treated successfully. We report this case as it is rare and to illustrate the technical aspect of surgical repair.

## Case presentation

**Figure 1:**
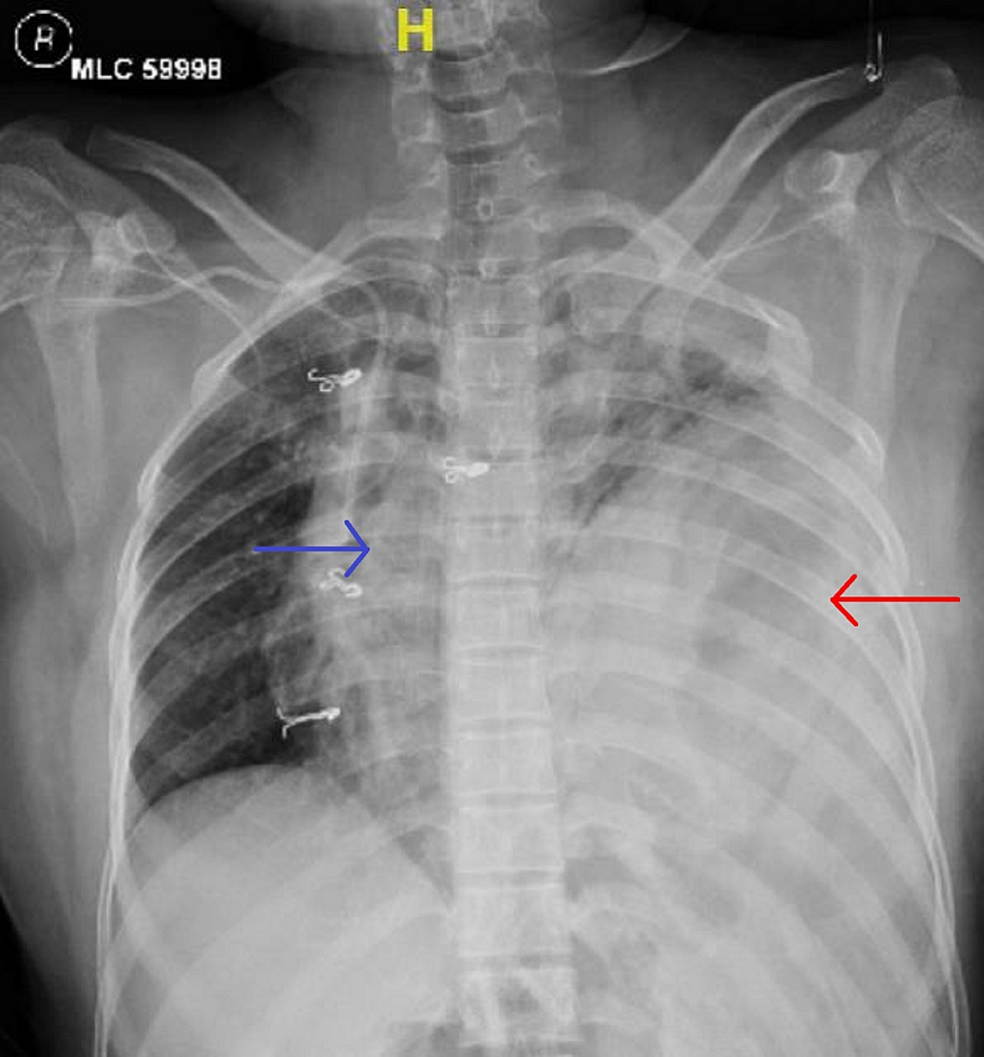
Chest X-ray A blue arrow shows the mediastinal shift and a red arrow shows the herniated bowel in the left hemithorax.

A twenty-eight-year-old female patient presented to the emergency department with a history of road traffic accidents. She had difficulty in breathing. The patient was conscious, oriented, and dyspneic, with a 110/60 mmHg blood pressure. Room air saturation was 90%, which improved with minimal oxygen supplementation. She had pain over the left side of the chest and abdomen, and there were reduced breath sounds on the left side of the chest. X-ray of the chest revealed herniation of the bowel in the left hemithorax and mediastinal shift to the right side (Figure 1).

**Figure 2:**
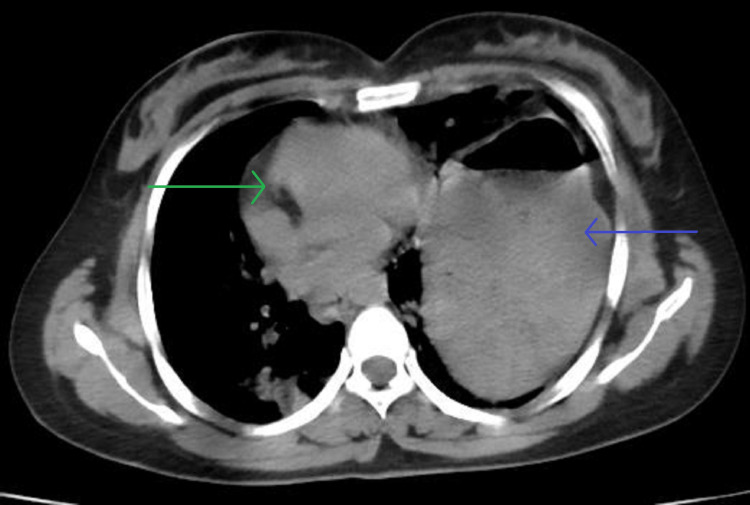
CT Thorax The blue arrow shows the herniated stomach in the left hemithorax, and the green arrow shows the mediastinal shift.

**Figure 3:**
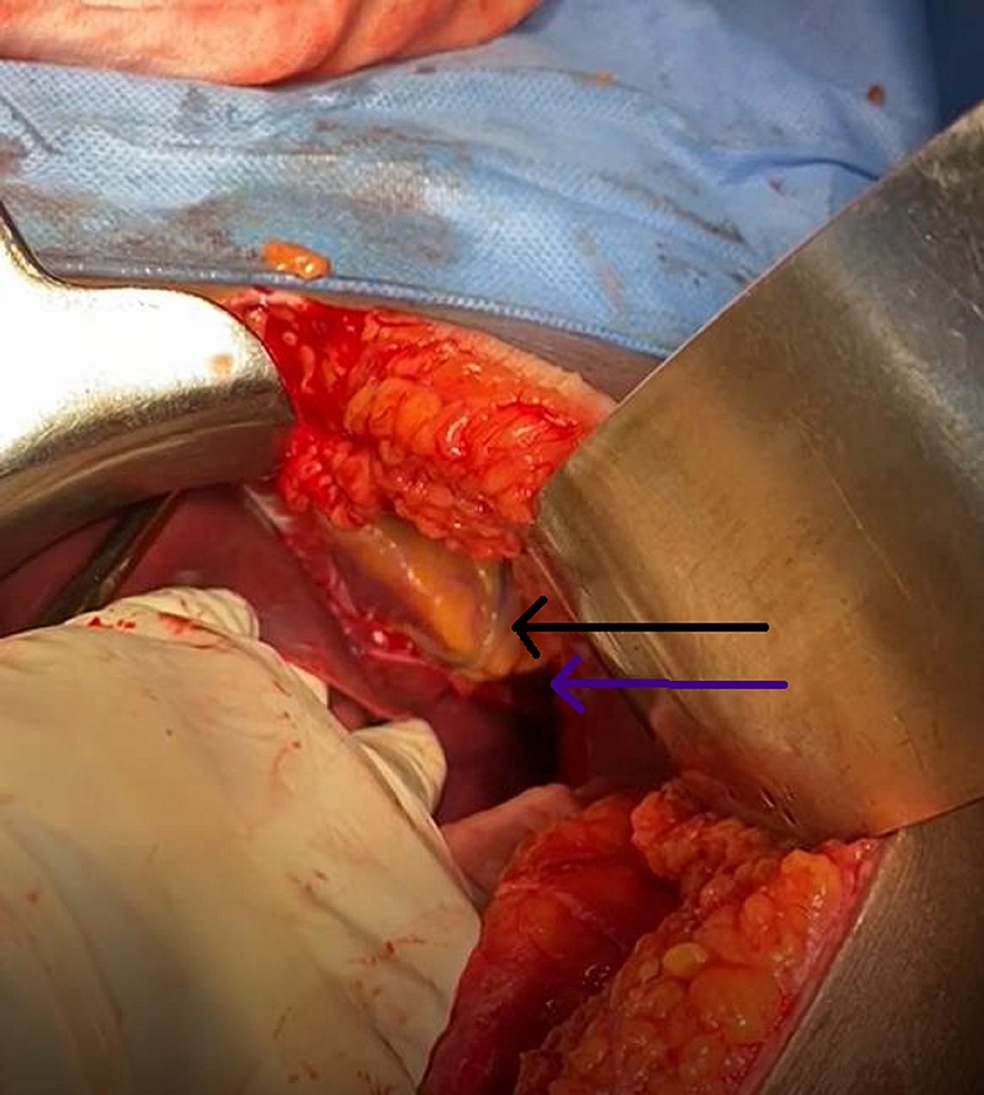
Intraoperative picture. Blue arrow showing the ruptured diaphragm. The black arrow point toward the right ventricle.

**Figure 4:**
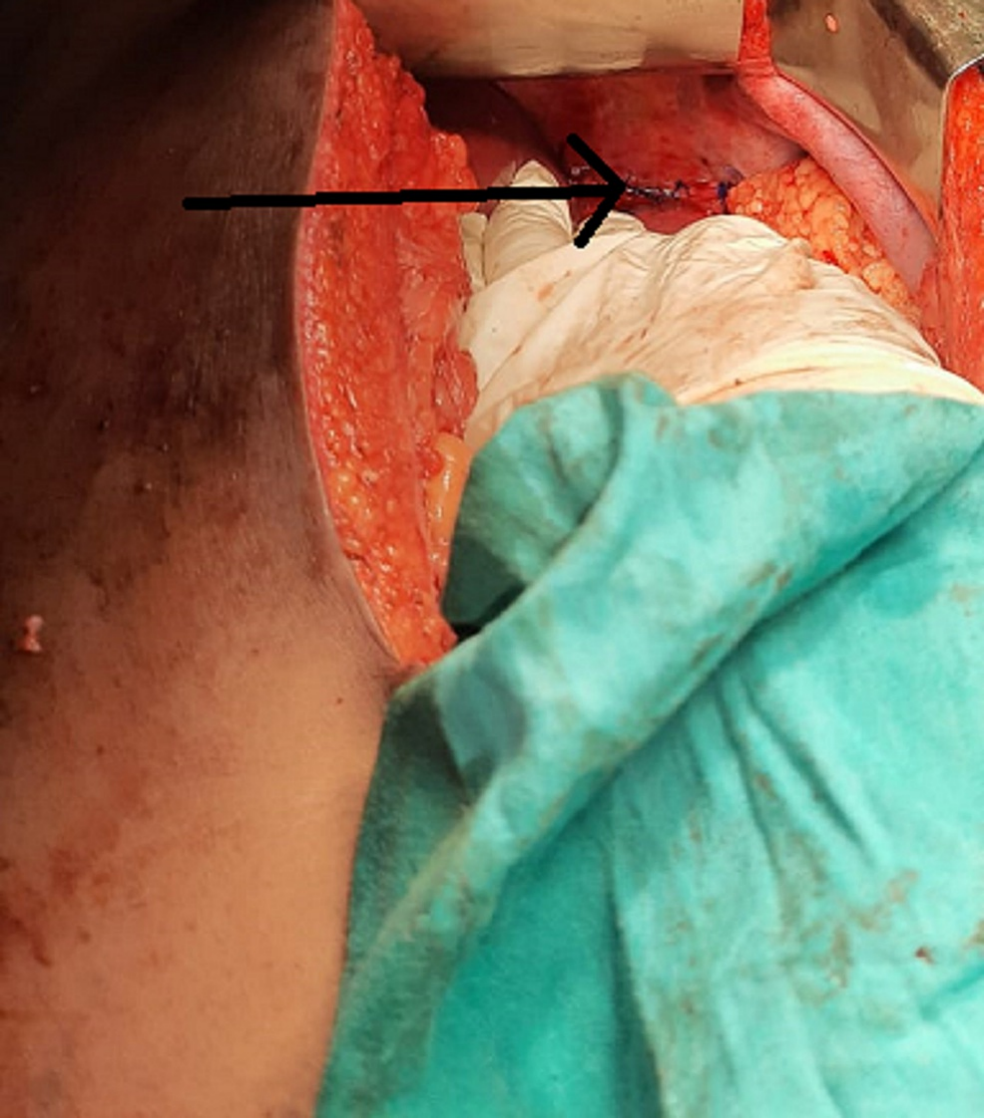
Intra-operative image. Black arrow showing the closed diaphragm (interrupted prolene sutures).

A ryles tube was placed, and the patient was stabilized. Computed tomography (CT) chest and abdomen showed diaphragmatic rupture with herniation of the stomach through the left hemidiaphragm (Figure 2). The patient also had a grade one splenic sub-capsular hematoma. She was taken up for emergency surgery. The abdomen was opened through a midline incision. There was minimal hemo-peritoneum, a large defect in the left hemidiaphragm, and pericardial rupture on the inferior aspect. The stomach and part of the small bowel were herniated into the left hemithorax. The bowel was reduced back to the abdomen (Figure 3). Interrupted 1-0 prolene sutures were taken, starting from the inferolateral part of the diaphragmatic cut surface and proceeding toward the pericardium. Care was taken while placing sutures in the pericardium to avoid injury to the right ventricle (Figure 4). Saline testing was done to confirm no air leak and ensured no connection with the thoracic cavity. The splenic hematoma was managed conservatively. The left Inter-coastal drain was placed. There was no other solid organ injury, and the abdomen was closed in layers. She was extubated the next day after surgery. The initial drain was minimal, and the drain was removed after extubation. Low-molecular-weight heparin was given for three days for deep vein thrombosis prophylaxis. Started ambulating on day five with support. She followed up after three months, and she was fine. Her lab reports were within normal limits.

## Discussion

Diaphragmatic ruptures following trauma are rare, accounting for less than 0.5% of all trauma and about 1.9% of all blunt abdominal trauma [4,5]. As in this case report, the left hemidiaphragm is more commonly involved than the right. The right hemidiaphragm is congenitally strong and well-protected by the liver [6]. Diaphragmatic ruptures are very difficult to diagnose, and they are usually associated with solid organ injury, and more often, the spleen is involved, as in this case report. Pericardial involvement and diaphragmatic rupture are extremely rare and are usually associated with an increased mortality rate ranging from 30-64% [7]. The hypothesized cause for this injury is increased intra-abdominal pressure following blunt abdominal trauma and the high-pressure difference between intra-abdominal and intra-thoracic cavities resulting in diaphragmatic rupture [8].

Patients usually present with breathing difficulty and abdominal pain. Few patients do not have any symptoms at the time of presentation, and they gradually worsen due to respiratory distress caused by bowel herniation into the thoracic cavity. Some patients have palpitations, chest discomfort, cardiac tamponade, or cardiogenic shock due to cardiac compression [9]. The patient was tachypneic and had breathing difficulty in this case.

Diaphragmatic hernia can be seen in chest X-ray as bowel in the thoracic cavity, loss of diaphragm contour with mediastinal shift to the contralateral side like in this case. The sensitivity of Multi-detector Computed Tomography (MDCT) in identifying diaphragmatic rupture is 100% and 83 % for the left side and right side diaphragmatic rupture, respectively [4]. Though Magnetic Resonance Imaging is more accurate, the duration of the examination is long [10].

Surgical repair remains the mainstay of treatment for pericardio-diaphragmatic rupture. Surgery can be done through laparotomy, thoracotomy, or laparoscopy. It involves reducing herniated content in the abdomen and repairing the diaphragm with non-absorbable sutures [11]. Other associated abdominal and visceral injuries can be ruled out when approached through the abdomen. Care should be taken while taking sutures over the pericardial layer to avoid injuring the heart. Combined thoracotomy and transabdominal approach can be useful in chronic diaphragmatic ruptures as adhesions between the pleural cavity, and bowel can be released carefully [6].

## Conclusions

Diaphragmatic rupture with pericardial involvement is rare and carries a poor prognosis. Pre-operative diagnosis of the pericardial injury is complicated, and CT is mandatory in diagnosing pericardial tear and excluding other visceral injuries. Care should be taken while closing the pericardium to avoid injuring the heart. Emergency surgical repair and close postoperative monitoring are always required to avoid severe complications.
